# CS1 Expression Pattern in NK Cells and Correlated Factors in Plasma Cell dyscrasias: Implications for Elotuzumab Therapy and CAR-T Efficacy

**DOI:** 10.7150/jca.93637

**Published:** 2024-04-08

**Authors:** Chunhui Li, Di Wang, Yanjie Xu, Xia Mao, Yimei Que, Zhe Li, Qiuxia Yu, Menglei Xu, Ning An, Xiaolu Long, Chunrui Li

**Affiliations:** 1 Department of Hematology, Tongji Hospital, Tongji Medical College, Huazhong University of Science and Technology, Wuhan, Hubei 430030, China.; 2 Immunotherapy Research Center for Hematologic Diseases of Hubei Province, Wuhan, Hubei 430030, China.

**Keywords:** CS1, Immunotherapy, Plasma cell dyscrasias, NK cells

## Abstract

Treatment with elotuzumab alone has no discernible antitumor effect and progress in chimeric antigen receptor T cells (CAR-T) therapy targeting CS1 is relatively slow. A retrospective analysis was performed on 236 patients with multiple myeloma (MM) and 30 patients with other plasma cell dyscrasias (PCDs). CS1 expression in NK cells, lymphocytes, and monoclonal plasma cells was assessed using multiparameter flow cytometry. Furthermore, new explorations were undertaken regarding the antitumor applications of elotuzumab. Patients with MM had significantly higher CS1 expression levels in plasma cells than other patients with PCDs, with no significant differences between lymphocytes and NK cells. In both patients with MM and other PCDs, CS1 expression was significantly higher in plasma cells than in NK cells and lymphocytes. Univariate and multivariate analyses revealed a significant correlation between CS1 expression in plasma (r = 0.60; P < 0.001) and NK (r = 0.79; P < 0.001) cells. Factors such as cytogenetic abnormalities, disease progression, and survival were not associated with CS1 expression in NK cells. Moreover, this study showed that elotuzumab strongly increases the cytotoxicity of NK cells against non-plasma and plasma tumor cells independent of their CS1 expression level. This underscores the potential of elotuzumab in combination with NK cells as an effective therapeutic strategy against a broad spectrum of tumor types.

## Introduction

CS1 is a member of the signaling lymphocyte activation molecule (SLAM) family that is widely expressed in human plasma and myeloma cells, making it a promising target for immunotherapy in multiple myeloma (MM). Elotuzumab, a monoclonal antibody that targets the CS1 protein, has been approved by the US Food and Drug Administration (FDA) for the treatment of relapsed/refractory MM 1. Although elotuzumab is effective when combined with lenalidomide or bortezomib 2-4, its antitumor efficacy is insufficient when used alone 5. Chinese and American clinical trial registration websites show that at least nine clinical trials focusing on chimeric antigen receptor T cells (CAR-T) targeting CS1 have been conducted since July 2014. Most of these trials are in Phase 1. There is a lack of clinical data available to date 6, 7, indicating the potential obstacles to CS1-targeted immunotherapy. CS1 is highly expressed in natural killer (NK) cells compared to other lymphocytes, and plays a vital role in CS1-mediated anti-tumor immunotherapy 8, 9. In this study, we examined the presence of CS1 on monoclonal plasma cells in several plasma cell diseases to evaluate the possibility of targeting CS1 in other plasma cell dyscrasias outside multiple myeloma. We also analyzed the CS1 expression pattern of CS1 on different cell types in plasma cell dyscrasias to explore new approach for CS1-related immunotherapy.

## Methods

### Patients

This retrospective study was approved by the Medical Ethics Committee of Tongji Medical College, Huazhong University of Science and Technology (Wuhan, China). The final follow-up date was April 15, 2023. Between January 2018 and September 2019, 266 patients were enrolled in this study, and flow cytometric analysis of CS1 expression was performed at our hospital. The patient population consisted of 236 patients with MM (175 newly diagnosed, 43 relapsed, and 18 undergoing treatment), 11 with systemic light chain amyloidosis (AL), 13 with monoclonal gammopathy of undetermined significance (MGUS), four with POEMS syndrome, and two with monoclonal gammopathy of renal significance (MGRS). Based on the expression of CS1 in NK cells, patients were divided into a high-expression group (CS1 MFI ≥ 75th percentile) and a low expression group (CS1 MFI ≤ 25th percentile).

Diagnoses of MM, MGUS, AL, and POEMS syndrome were confirmed according to the updated International Myeloma Working Group (IMWG) criteria 10. The diagnosis of MGRS was confirmed according to the International Kidney and Monoclonal Gammopathy Research Group (IKMG) diagnostic criteria 11.

### Flow cytometric analysis of CS1 expression

The level of membrane-bound CS1 expression was defined as CS1 expression intensity, which was determined by quantitative flow cytometry based on the mean fluorescence intensity (MFI). Surface antigen CS1 expression was detected using multiparameter flow cytometry (FACSCanto, Becton Dickinson). BD FACSDiva 8.0.2 software was used for the data analysis. Fluorochrome-conjugated antihuman antibodies were purchased from BD Biosciences (Basel, Switzerland). Peripheral blood mononuclear cells (PBMCs) were isolated from the patient's whole blood, and mononuclear cells were collected from the PBMCs to obtain lymphocytes. Malignant monoclonal plasma cells can be recognized by the presence of the markers CD45dim/^-^, CD38^+^, CD138^+^, CD19^-^, and CD56^+^. If plasma cells do not express CD56, additional markers such as clambda/ckappa, CD20, and CD117 are required. In addition, the expression of CS1 in lymphocytes was examined. CD16^+^/CD56^+^/CD45^+^/CD3^-^ NK cells were detected and their CS1 expression was examined. As this was a retrospective clinical study, the gating of lymphocytes inherently included NK cells. Figure S1 shows the comprehensive gating technique.

### Cells and cell lines

Blood was collected from healthy adult donors, and PBMCs were isolated using Ficoll-Paque PLUS (GE Healthcare) according to the manufacturer's instructions. Monocytes were depleted from the PBMCs using CD14 microbeads (Miltenyi Biotec, Auburn, CA) or CD14 positive selection kit (StemCell Technologies), and the preparation was confirmed to be >90 % depleted of monocytes. PBMCs depleted of monocytes (PBL-enriched) were used for the assays. NK cells were enriched from PBMCs using EasySep® NK cell negative selection kit (StemCell Technologies) and confirmed to be >90 % pure by flow cytometry for CD56. The Nalm-6, Raji, RPMI8266, and U266 cell lines were obtained from ATCC. The Lenti-luciferase fluorescent protein (luci) transfection vector (Invitrogen) was used to generate Nalm-6-, Raji-, RPMI8266-, and U266-luci cells following the manufacturer's instructions.

### Reagents

Humanized anti-CS1 antibody (elotuzumab) was obtained from Selleck Chemicals (Shanghai, China). Anti-human CD319 (SLAMF7, CRACC: clone 162.1)-APC, anti-CD56 (HCD56)-PE, and anti-107a-APC were purchased from BioLegend.

### NK cell degranulation assay

NK cells were incubated with elotuzumab (100 µg/mL) or IgG1 (100 µg/mL) for 24 h at 37 °C, 5% CO_2_, and washed to remove unbound and excessed antibodies. NK cells were then co-cultured with target cells for 4 h at 37°C, 5% CO_2_. Cells were stained with anti-CD56-PE and anti-CD107a-APC antibodies (BioLegend). The percentage of CD56^+^CD107a^+^cells was determined using flow cytometry. To detect CD107a by direct engagement on NK cells, these cells were incubated either alone or with elotuzumab (100 µg/mL) for 24 h at 37°C, 5% CO_2_. Cells were stained with anti-CD56-PE and anti-CD107a-APC antibodies (BioLegend). The percentage of CD56^+^CD107a^+^ cells was determined using flow cytometry.

### Cytotoxicity assay

NK cells were seeded into 96-well plates at a density of 50,000 cells/well and were incubated with elotuzumab (100 µg/mL) for 24 h at 37 °C, 5% CO_2_, and washed to remove unbound antibodies. Next, NK cells were co-cultured with Nalm-6-, Raji-, RPMI8266-, and U266-luci cells at effector-to-target (E:T) ratios of 1:2 and 1:1 (200 µL total volume/well) for another 24 h. The plate was washed again with PBS, and 100 µL of luciferin at 150 μg/mL was added to each well. The fluorescence intensity in each well was promptly measured using Biotek synergy2 (Biotek, USA).

### Statistical analysis

Categorical variables were described as frequencies (percentages), parametric data were presented as means (± standard deviation, SD), and nonparametric data were described as medians (ranges). The Mann-Whitney U test was used to compare quantitative variables between two groups, and the Kruskal-Wallis test with Dunn's multiple comparison test was used for three or more groups. The D'Agostino-Pearson normality test was performed to check the data distribution. Correlation analysis was performed using Spearman's correlation test. Multivariate linear regression analysis was used to analyze the association between the clinical parameters and CS1 expression. Progression-free survival (PFS) was defined as the period from the time of diagnosis to second-line treatment or treatment discontinuation. For patients with relapse, PFS was calculated from the current treatment initiation, at which point CS1 was determined, until the next confirmed disease progression or death owing to disease progression. Overall survival (OS) was defined as the period from the date of diagnosis until death or last follow-up. PFS and OS curves were estimated using the Kaplan-Meier analysis, and statistical differences between the groups were compared using the log-rank test. The association between survival and prognostic factors was determined using multivariate analysis using the Cox proportional hazards model. All tests were two-tailed, and a P-value of less than 0.05 was considered statistically significant. The statistical significance of the *in vitro* results was analyzed using a two-tailed unpaired Student's t-test. All experiments were performed with biological replicates; that is, measurements were taken from distinct samples. Statistical analyses were performed using SPSS version 27.0 software (IBM Corp, Armonk, N.Y., USA) and GraphPad Prism version 9.5.

## Results

### CS1 expression in NK cells, lymphocytes, and clonal plasma cells

**Figure 1:**
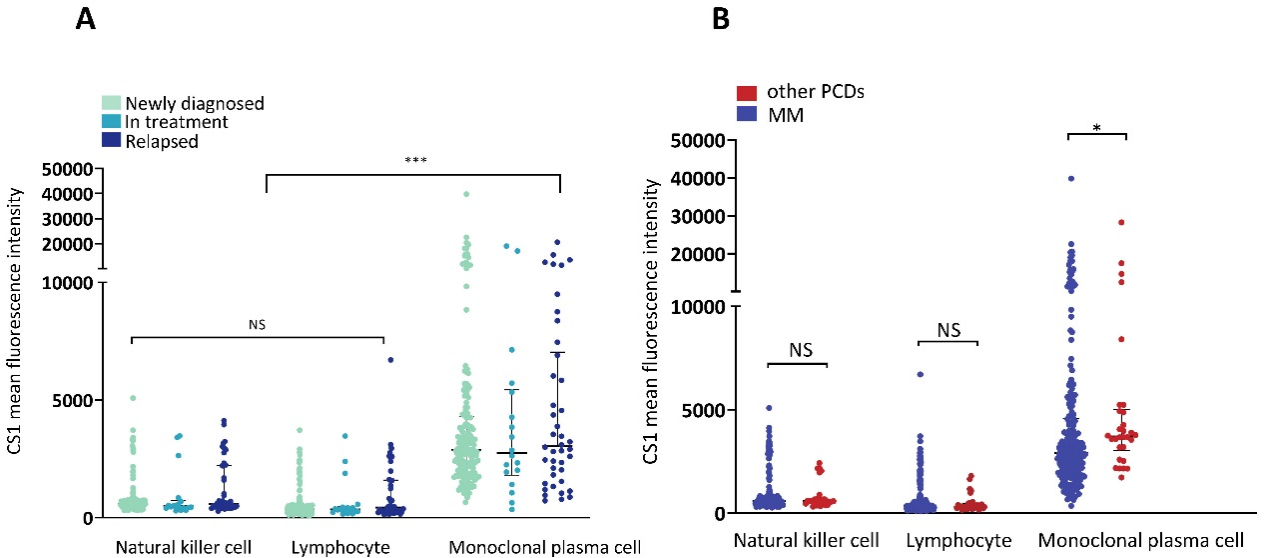
** Expression of CS1 in NK cells, clonal plasma cells, and lymphocytes. (A)** The expression of CS1 changed in newly diagnosed, under-treated, and relapsed patients with MM. There were no statistically significant differences between the CS1 MFI in patients at different disease stages. The median CS1 MFI of NK cells in patients at newly diagnosed, in treatment, and relapsed phases were 589 (304-5086), 501.50 (297-3479), and 590 (274-4128), respectively. The median CS1-MFI of lymphocytes was 357 (91-3718), 352.50 (157-3474), and 453 (154-6707), respectively. The median CS1-MFI of clonal plasma cells was 2893 (659-39801), 2751 (352-18992), and 3047 (723-20552), respectively. **(B)** Bar graphs show CS1 MFI of NK cells, lymphocytes, and plasma cells in patients with MM or other PCDs. In patients with MM or other PCDs, clonal plasma cells had significantly higher CS1 expression levels than NK cells and lymphocytes. No significant difference in CS1 expression levels was observed between NK cells and lymphocytes. Patients with MM had a significantly higher CS1 MFI of clonal plasma cells than patients with other PCDs [2910 (352-39801) vs. 3714.50 (1727-28271), P=0.014]. There was no difference between the expression levels of CS1 in NK cells and other lymphocytes in these two groups.

**Figure 2:**
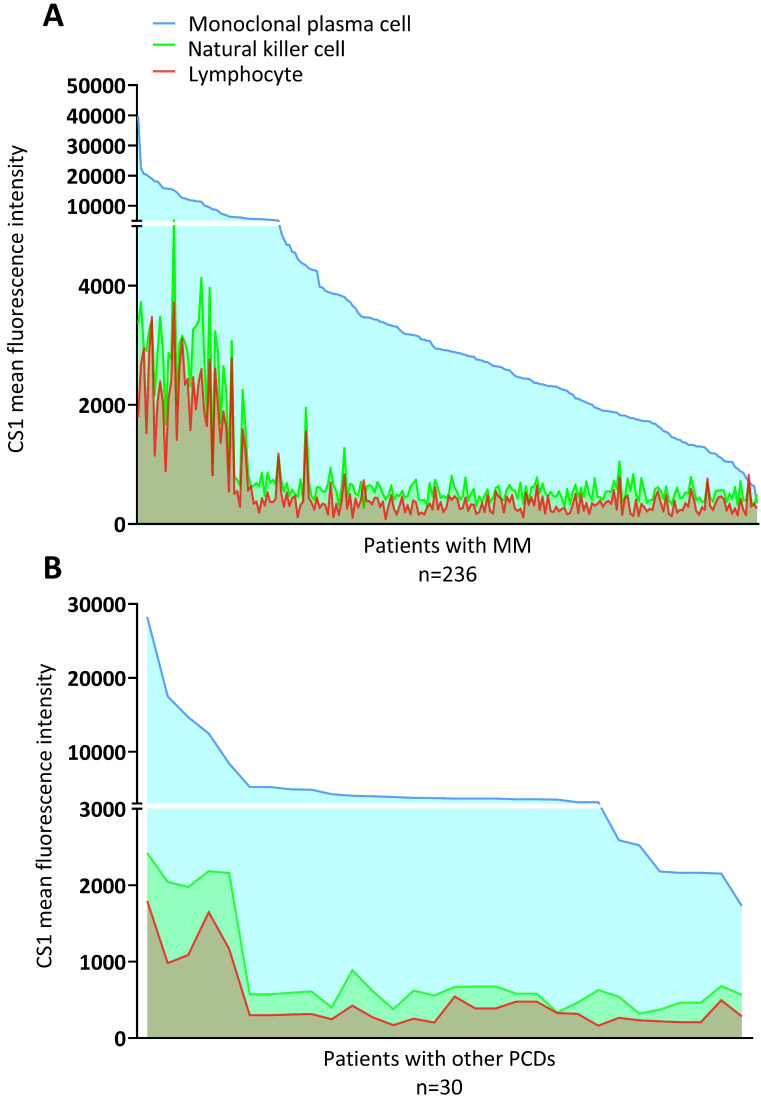
** Expression of CS1 in the three cell subtypes within a single individual. (A)** The level of CS1 expression by NK cells, plasma cells, and lymphocytes in patients with MM. In patients with MM, the median CS1 MFI of clonal plasma cells, NK cells, and lymphocytes were 2910 (352-39801), 581 (274-5086), and 366 (91-6707), respectively. **(B)** The level of CS1 expression by NK cells, plasma cells, and lymphocytes in patients with other PCDs. In patients with other PCDs, the median CS1 MFI of clonal plasma cells, NK cells, and lymphocytes was 3714.50 (1727-28271), 588.50 (320-2422), and 312 (164-1797), respectively.

The MGUS, MGRS, AL, and POEMS syndromes were grouped with other PCDs. The clinical characteristics of the patients with MM and other PCDs are presented in Table 1. There were no significant differences in the expression of CS1 in NK cells or lymphocytes between the two groups (Figure 1A). However, patients with MM had significantly higher levels of CS1 expression in plasma cells compared to patients with other PCDs. Notably, CS1 was consistently expressed in all three cell types in patients with MM regardless of the disease state (Figure 1B). The expression of CS1 in the three cell subtypes of each patient is shown in Figure 2A-2B. In both MM (Figure 2A) and other PCDs (Figure 2B), plasma cells showed significantly higher CS1 expression than NK cells and lymphocytes. NK cells exhibited a slightly higher CS1 expression than lymphocytes; however, this difference was not statistically significant. Moreover, the CS1 expression levels in these three cell subsets showed an apparent positive correlation.

### Correlation of CS1 expression levels in NK cells with clinical parameters

**Figure 3:**
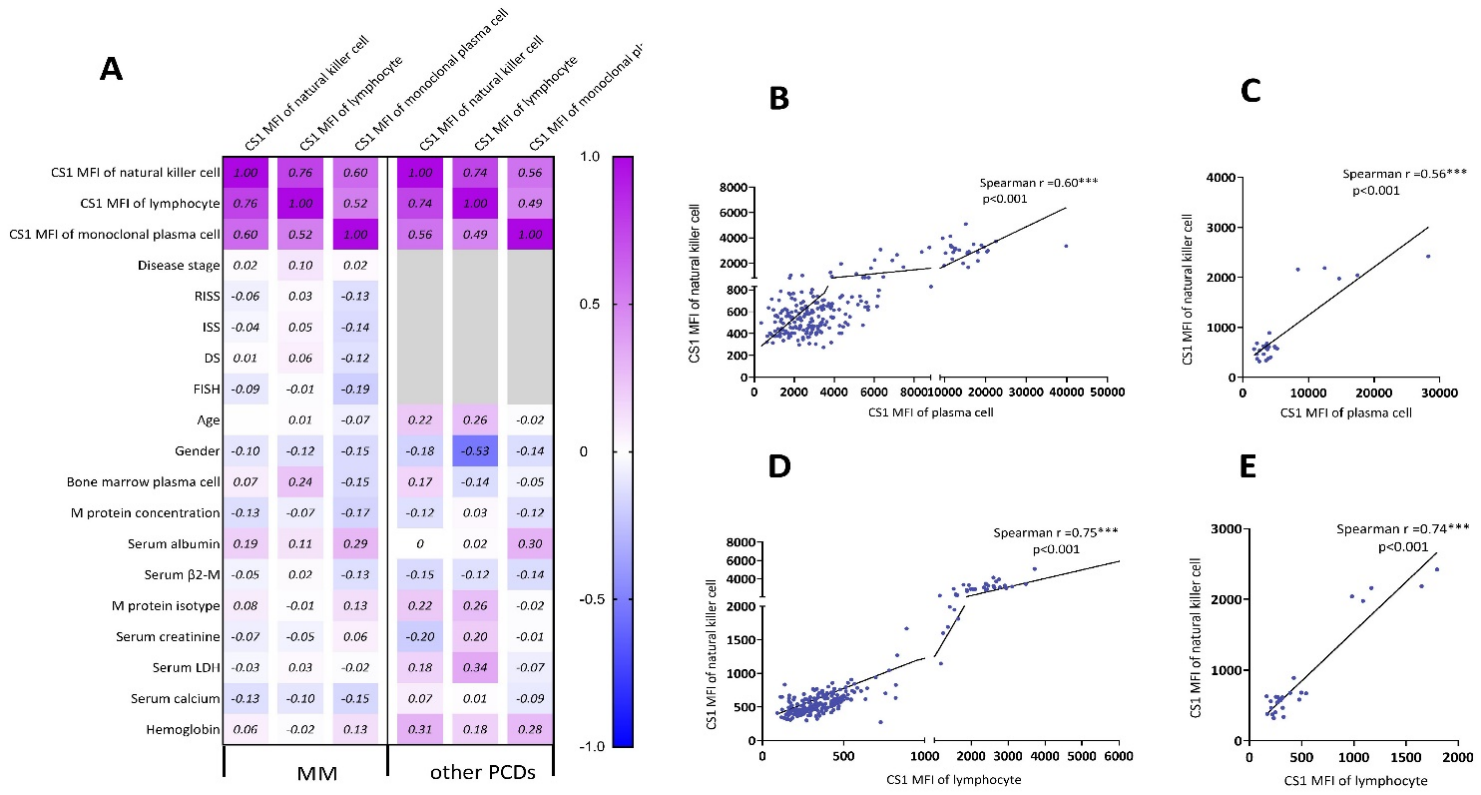
** Spearman correlation analysis of CS1 expression on NK cells and clinical parameters. (A)** Sex, age, hemoglobin, albumin, serum calcium, serum creatinine, monoclonal protein concentration, β2-M, LDH, FISH, M protein isotype, disease stages (newly diagnosed, in treatment, and relapsed phases), and plasma cells in bone marrow were some of the clinical parameters we examined using Spearman correlation analysis to determine the relationships between CS1 expression levels. **(B-E)** The correlations were shown with statistically significant p-values (< 0.05). (B) and (C) show the Spearman relationships between CS1 MFI of plasma cells and NK cells in MM and other PCDs, respectively. (D) and (E) show Spearman correlations between the CS1-MFI of NK cells and the CS1-MFI of lymphocytes in MM and other PCDs, respectively.

**Figure 4:**
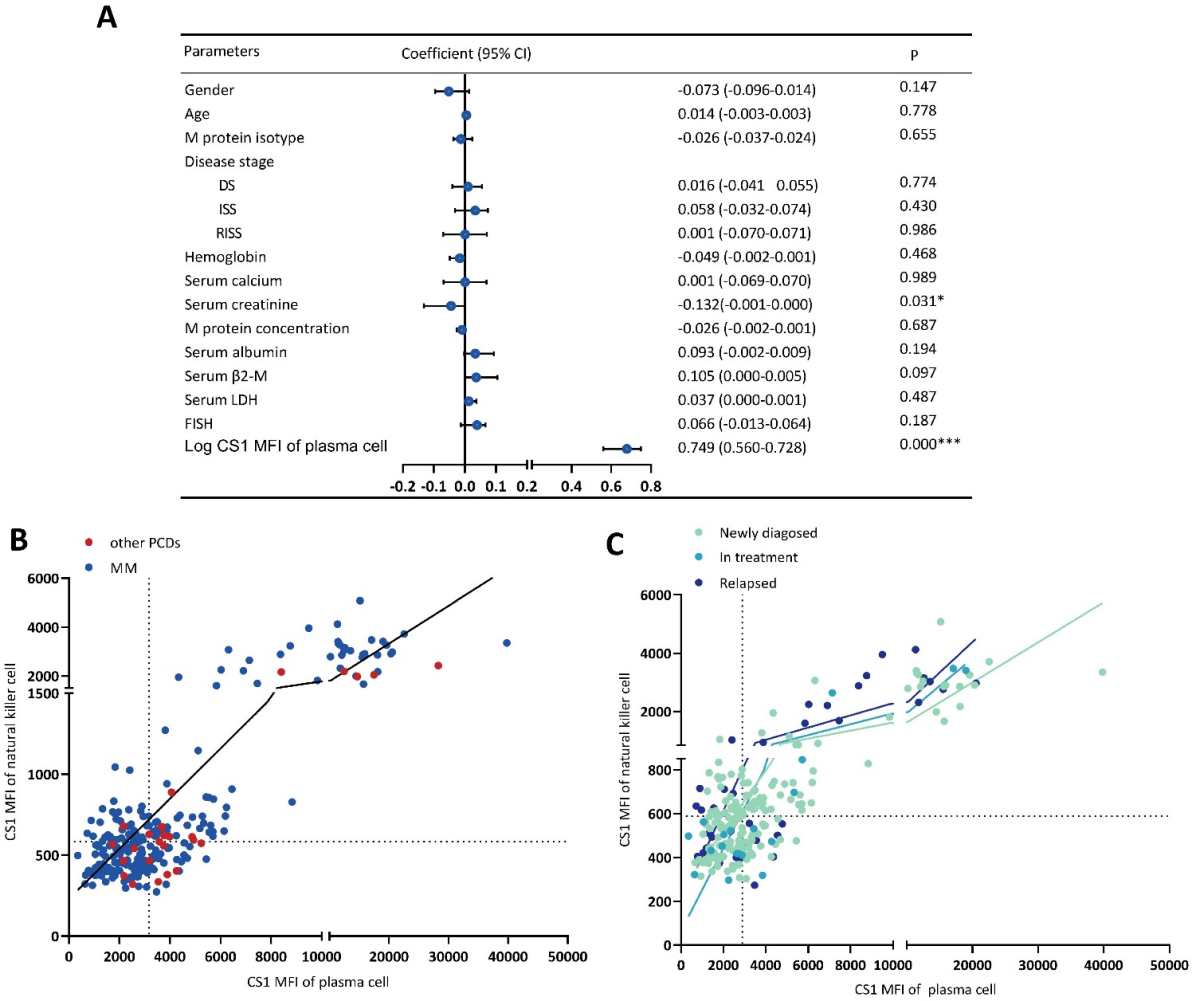
** CS1 expression levels in NK cells are significantly associated with CS1 expression levels in clonal plasma cells. (A)** Forest plot showing the correlation between CS1 expression levels in NK cells and clinical parameters by multivariate linear regression analysis. **(B, C)** Overview of CS1 expression in NK cells and clonal plasma cells. The scatter plot shows CS1 expression in NK cells and plasma cells according to CS1 MFI. It is divided into four quadrants based on the median lines. Different dotted colors indicate different populations. (B) CS1 expression in NK and plasma cells was observed in all patients. (C) CS1 expression in NK and plasma cells at different stages of MM.

The relationship between the clinical parameters and CS1 expression is shown in Figure 3A. The CS1 expression level in NK cells was significantly associated with that in plasma cells (Figure 3B-3C) and lymphocytes (Figure 3D-3E) present in patients with MM or other PCDs. Before performing multivariate linear regression analysis to determine independent associations between CS1 expression in NK cells and clinical factors, the CS1 MFI value was log10-transformed. CS1 expression in NK cells (r = 0.749; P < 0.001) also showed a positive correlation with the log CS1 MFI in plasma cells (Figure 4A). Of note, CS1 expression levels of NK cells were not associated with the other clinical factors, including sex, age, hemoglobin, albumin, serum calcium, serum creatinine, serum β2-microglobulin (β2-M), lactic dehydrogenase (LDH), different disease stages, M protein isotype and concentration, or high-risk genetic abnormalities. Interestingly, elevated baseline serum albumin levels were positively correlated with plasma cell CS1 expression (r = 0.29; P < 0.001) in patients with MM. The CS1 MFI of lymphocytes was also positively associated with that of plasma cells in both patients with MM and other PCDs (Figure S2). We performed the same multifactorial analysis on plasma cells and found that the expression levels of CS1 in plasma cells positively correlated with the log CS1 MFI of NK cells (r =0.737; P < 0.001) (Figure S3). As NK cells are naturally present in lymphocytes, further research is necessary to ascertain the true relationship between NK cells and other lymphocytes. Therefore, we focused on the correlation between CS1 expression levels in NK and plasma cells. An overview of CS1 expression in NK and monoclonal plasma cells is shown in Figure 4B-4C to illustrate this relationship more clearly. In the majority of patients located on the left lower and right upper quadrants, a strong positive relationship between CS1 expression levels in plasma and NK cells is evident (Figure 4B). As shown in Figure 4C, this positive relationship remained unchanged at different disease stages.

### Correlation of CS1 expression levels in NK cells with patient survival

**Figure 5:**
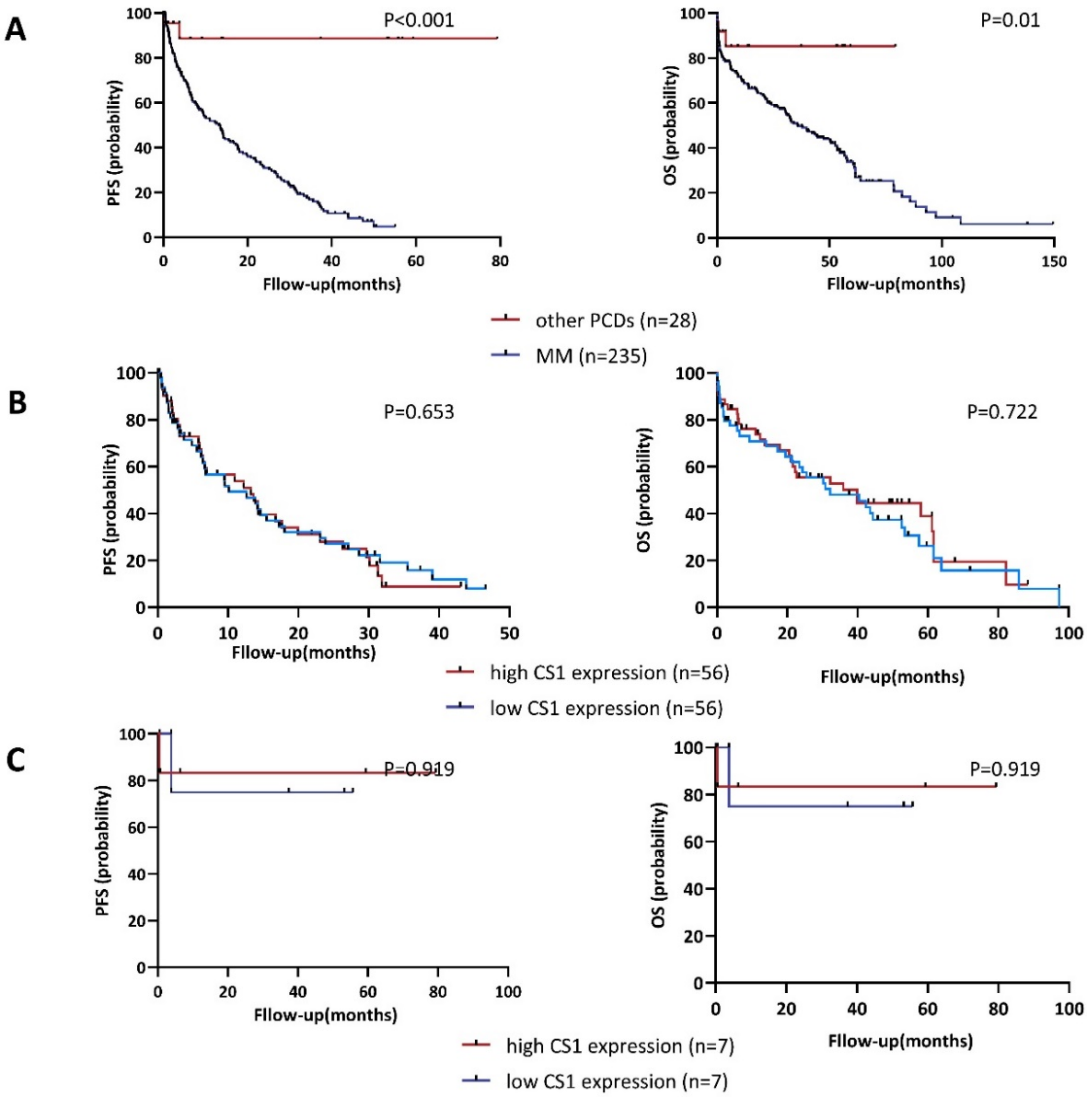
** Correlation of CS1 expression levels in NK cells with PFS and OS. (A)** Kaplan-Meier estimates of OS and PFS in patients with MM (n=235) and other PCDs (n=28). Patients with MM had a median OS and median PFS of 21.071 (0-149.429) and 5.367 (0-55.036) months, respectively. For patients with other PCDs, they were 3.821 (0-80.536) and 6.393 (0-80.536) months, respectively. **(B-C)** According to the expression of CS1 on NK cells, patients were divided into a high expression group (CS1 MFI ≥ 75th percentile) and a low expression group (CS1 MFI ≤ 25th percentile). (B) Comparative analysis of OS and PFS between patients with MM and high and low CS1 expression of NK cells. (C) Comparative analysis of OS and PFS between patients with other PCDs and high and low CS1 expression of NK cells.

As of April 2023, the median follow-up was 22.36 (0.04 - 149.43) months and 5.13 (0.36 - 79.32) months for patients with MM (n = 235) or other PCDs (n = 28), respectively. We examined the distribution of OS and PFS between the high CS1 expression group and the low expression group to assess whether the CS1 expression levels in NK cells could be a useful indicator of survival. As shown in Figure 5A, patients with PCD had significantly longer OS and PFS than those with MM. In addition, we found no significant difference in survival outcomes between the high CS1 expression group and the high CS1 expression group in patients with MM (Figure 5B) or other PCDs (Figure 5C). Furthermore, we found no correlation between plasma CS1 levels and survival outcomes (Figure S4). Consistent with this observation, multivariate Cox analysis of PFS and OS revealed that neither CS1 expression levels in NK cells nor plasma cells were independent predictors of survival (Table S1).

### Integrating CS1 expression in NK cells for novel therapeutic approaches

**Figure 6:**
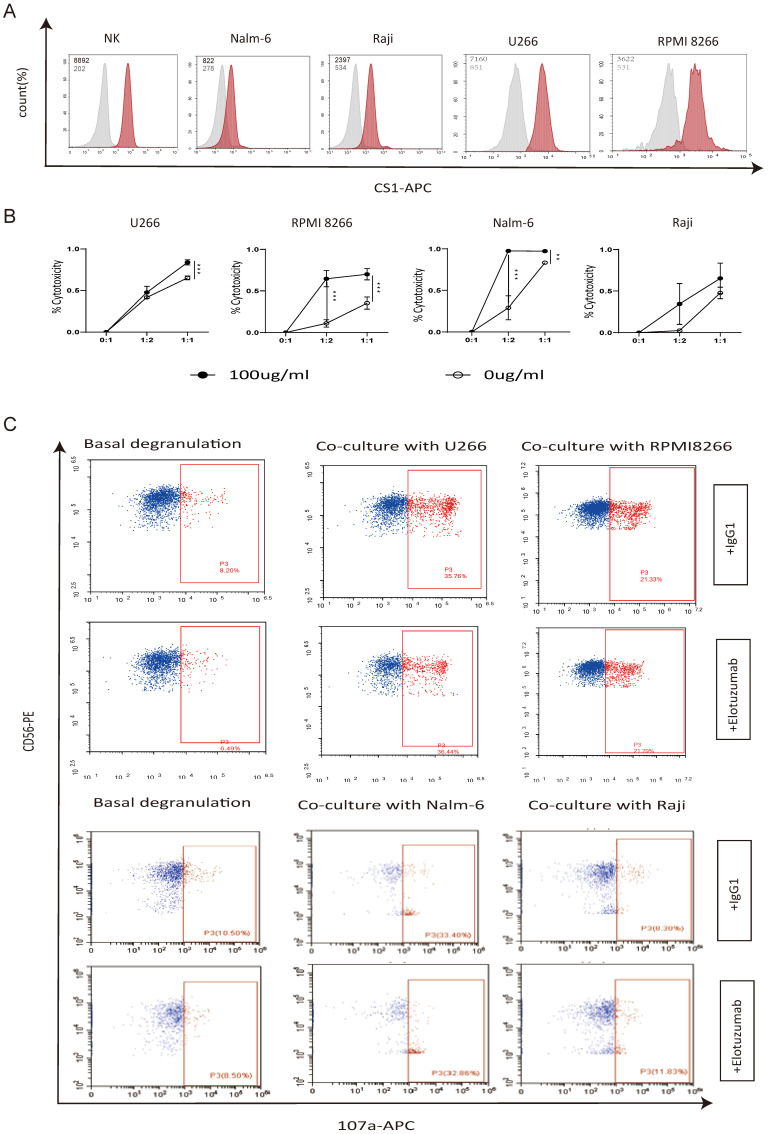
** Elotuzumab enhances the cytotoxicity of NK cells independent of the degree of CS1 expression on target cells. (A)** Flow cytometry was used to evaluate the expression of CS1 on normal NK cells from a healthy donor, as well as Nalm-6, Raji, RPMI 8266, and U266 cells. MFI values are displayed in the upper corner. **(B)** NK cells were exposed to 100 µg/mL of elotuzumab for 24 h. Subsequently, they were co-cultured with target cells at effector-to-target (E:T) ratios of 1:1 and 1:2 for another 24 h (n=3). The luciferase signal was subsequently quantified using Biotek Synergy2. **(C)** Elotuzumab does not increase the CD107a expression on NK cells when co-cultured with Nalm-6, Raji, RPMI 8266, and U266 cells. NK cells were treated with either 100 μg/mL of IgG1 or elotuzumab and then co-culture with target cells at effector-to-target (E:T) ratios of 1:1 for 4 h (n=3). The percentage of CD107a-positive NK cells was assessed using flow cytometry. Significance levels: * P<0.05, ** P<0.01, *** P<0.001.

To explore the potential use of elotuzumab to enhance the ability of autologous NK cells to eliminate tumor cells that exhibit negative or low CS1 expression levels, we initially assessed the expression levels of CS1 on NK cells, myeloma cell lines (RPMI 8266 and U266), and non-plasma cell tumor cell lines (Nalm-6 and Raji). Our findings, depicted in Figure 6A, indicate that CS1 expression levels on myeloma cell lines were significantly higher compared to those on non-plasma cell tumor cell lines. In our experiments (Figure 6B), we found that elotuzumab alone had no significant effect on the activity of tumor cells, and the cytotoxicity of NK cells alone against tumor cells was limited. However, NK cells treated with elotuzumab exhibited a significantly enhanced killing effect on tumor cells, regardless of whether the target cells expressed CS1. Additionally, we investigated the expression of CD107a, a marker of NK cell activation and cytotoxic activity, to determine whether elotuzumab influences functional NK cell markers 12. Figure 6C shows that CD107a degranulation in NK cells remained unchanged when grown with myeloma or non-myeloma cells.

## Discussion

The primary treatment method for other PCDs involves the elimination of monoclonal plasma cells using therapeutic protocols similar to those used for MM. Thus, CS1 is a crucial target in MM therapy and should be further investigated for its effectiveness in the treatment of other PCDs. We studied the presence of CS1 in monoclonal plasma cells of patients with different plasma cell dyscrasias to determine whether CS1-targeted immunotherapy could be used beyond MM. Our results showed that CS1 expression was less prominent in these illnesses than in MM, but was still significant. Unlike previous studies that found no significant differences in CS1 expression levels between MM other PCDs 13, 14, our results suggest that the disparities observed may result from the different detection methods employed: compared to immunohistochemistry and DNA-level analysis, flow cytometry provides more objective description of CS1 expression. Furthermore, we found no association between CS1 expression in monoclonal plasma cells and cytogenetic abnormalities, disease advancement, or survival rates, indicating that CS1 is a promising target for other PCDs. No differences in CS1 expression in NK cells were observed between MM and other PCDs. Therefore, we believe that immunotherapies targeting CS1, such as elotuzumab-based combination therapy and CS1 CAR-T, can be expanded to address a broader spectrum of plasma cell dyscrasias. In fact, initial studies have shown positive results for CS1 CAR-T and elotuzumab in the treatment of AL amyloidosis 13, 15.

We found a significant positive correlation between CS1 levels in NK and monoclonal plasma cells. This correlation is important for both CS1 CAR-T therapy and elotuzumab treatment. Despite being approved to treat RRMM, elotuzumab demonstrates a suboptimal antitumor efficacy when used alone 16, 17. Compared to elotuzumab, anti-CD38 and anti-CD20 monoclonal antibodies have multiple pathways of action, both of which include antibody-dependent cellular cytotoxicity (ADCC), complement-dependent cytotoxicity (CDC), and direct antitumor effects triggered by antibody-target antigen binding 18, 19. Elotuzumab itself has inadequate anti-myeloma activity and relies on NK cells to trigger target cell lysis by limited mechanisms 20, and when the number of NK cells is reduced, the therapeutic efficacy of elotuzumab is significantly lower (by ∼60%)^14^. It acts primarily through ADCC but also directly activates and promotes NK cell function and disrupts CS1-mediated adhesion of MM cells to bone marrow stromal cells 21. Given the pivotal role of NK cells in elotuzumab therapy, it is imperative to assess their quantity and quality to ensure the efficacy of the treatment. To enhance the efficacy of elotuzumab monotherapy, certain strategies such as augmenting NK cell quantity and activity should be employed (Figure S5).

Unlike elotuzumab, CS1 CAR-T directly destroy CS1-positive cells through cytotoxicity 22, 23. However, NK cells expressing CS1 induce on-target, off-tumor toxicity 24, thereby diminishing the safety and potency of the cell product. Nevertheless, NK cells expressing relatively low levels of CS1 can be killed by CS1 CAR-T, with the risk of excessive toxicity, because the antigen density on target cells required for CAR recognition is much lower than that required for antibodies 25. NK cells play an important role in CS1-mediated immunity during anti MM therapy. Therefore, killing NK cells with CS1 CAR-T not only leads to excessive toxicity but also weakens the immunological anti-MM effect of NK cells (Figure S5). Moreover, we found a significant positive correlation between CS1 expression in NK and clonal plasma cells. Therefore, it is necessary to mitigate the effect of CS1 expression on NK cells during CS1 CAR-T therapy. Previous research 26 acknowledged T-cell fratricides and constructed tandem CAR-T targeting CS1 and BCMA, effectively eliminating the fratricidal effects caused by CS1 expression on normal lymphocytes. Concerns regarding the CAR-T fratricide have been raised by several CAR-T manufacturing techniques 6, 27, such as the production of CS1 CAR-T derived from lymphocytes with a negative CS1 phenotype and the removal of CS1 expressed on T cells before the CAR structure is introduced. Nonetheless, our study serves as a cautionary note for clinicians involved in the clinical application of CS1 CAR-T therapy. This underscores the importance of paying particular attention to the levels of CS1 on the surface of NK cells because of the positive correlation between CS1 levels in NK cells and CS1 levels in tumor cells. This awareness is crucial for optimizing therapeutic outcomes and minimizing undesired effects in clinical settings.

CS1 is expressed in mature NK cells and plays an important role in NK cell activation 28-31. CS1 induces cytokine secretion by NK cells by recruiting EWS-activated transcript-2 (EAT-2), activating PI3K and phospholipase C signaling pathways, and killing target cells 28, 29. As CS1 is a self-ligand, NK cell function can be enhanced by CS1-CS1 interactions between NK or NK cells and their target cells 32. As previously mentioned, the expression of CS1 on plasma cells in other PCDs is significantly reduced compared to MM. Since CS1 is continuously and relatively highly expressed on NK cells, we thus looked into the use of elotuzumab to improve the activity of autologous NK cells for targeting tumours with low or negative CS1 expression. In our experiments, neither NK cells nor elotuzumab alone had a significant anti-tumor effect, but their combination exhibited an enhanced killing effect, regardless of whether the target cells expressed CS1**.** These initial results suggest the possibility of expanding the use of elotuzumab to treat a wider range of diseases by utilizing the properties and functions of CS1 expression in NK cells. Interestingly, despite the observed increase in NK cell cytotoxicity under the influence of elotuzumab, CD107a degranulation did not show any significant changes. This can be attributed to the diversity of cytotoxic mechanisms employed by NK cells 33, subtle regulatory adjustments in granule release mechanisms, differences in granule composition 34, limitations in detecting CD107a degranulation 35, and the influence of other cellular factors 36, 37. Thus, enhancing NK cell cytotoxicity is a multifactorial and complex process that is not directly reflected by changes in CD107a degranulation levels. Further in-depth studies are warranted to comprehensively assess the cytotoxic mechanisms of NK cells using various methodologies to gain a better understanding of their therapeutic potential.

## Conclusions

This study found that the levels of CS1 expression in clonal plasma and NK cells were consistent in PCDs and were not linked to cytogenetic abnormalities, disease progression, or survival outcomes. The correlation between CS1 levels in NK cells and CS1 levels in clonal plasma cells suggests possible implications for immunotherapy techniques aimed at targeting CS1. This highlights the importance of considering and managing the negative effects associated with NK cells expressing CS1 when employing CS1 CAR-T therapy. This study suggests a promising approach for utilizing elotuzumab to enhance the cytotoxicity of NK cells for the treatment of a broader spectrum of diseases.

## Supplementary Material

Supplementary figures and table.

## Figures and Tables

**Table 1:** Clinical characteristics of the patient population

Characteristic	MM					Other PCDs (30)
		Total (236)	ND (175)	In treatment (18)	Relapsed (43)	
Age, years						
Median (range)		60 (36-83)	61 (36-83)	61 (47-75)	54 (43-72)	53 (34-78)
Sex						
Male (n, %)		141 (60)	100 (42)	13 (72)	28 (68)	20 (67)
M protein isotype						
IgG		108	79	10	19	12
IgA		57	46	3	8	9
Light chain		55	39	0	11	5
IgD & IgE		10	6	0	4	0
others		6	5	0	1	3
Disease stage at study entry						
ISS						
l/ll/lll		41/76/119	28/66/81	5/4/9	8/6/29	——
R-ISS						
l/ll/lll		18/156/62	12/123/40	2/10/6	4/23/16	——
DS						
l/ll/lll		25/22/189	18/16/141	7/2/8	0/3/40	——
DS type						
A/B		190/46	136/39	3/15	4/39	——
Time since diagnosis, months						
Median (range)		21.07 (0.00-149.43)	10.64 (0.00-69.18)	35.93 (0.00-108.36)	42.42 (0.46-149.43)	3.82 (0.00-79.32)
Bone marrow plasma cells (%)	5.70 (0.02-79.00)		5.60 (0.02-79.00)	4.00 (0.35-76.00)	7.50 (0.03-75.20)	0.84 (0.16-3.50)
≤10		150	114	13	23	30
>10 to <30		56	46	2	8	0
≥30 to <60		25	8	2	10	0
≥60		5	2	1	2	0
Hemoglobin (g/L)						
Median (range)		87 (36-166)	87 (36-166)	97 (69-143)	83 (42-148)	126 (73-162)
LDH (IU/L)						
Median (range)		191 (57-1867)	188 (69-1495)	236.50 (126-1867)	201 (57-1867)	200 (101-613)
Creatinine(μmol/L)						
Median (range)		86.00 (2.21-1087.00)	88.00 (2.21-1087.00)	90.50 (41.00-887.00)	81 (36-341)	91 (43-618)
Calcium (mmol/L)						
Median (range)		2.46 (1.60-4.40)	2.48 (1.60-4.40)	2.47 (2.20-3.36)	2.43 (2.00-3.40)	2.37 (1.87-2.78)
Albumin (g/L)						
Median (range)		34.75 (15.90-50.30)	34.20 (15.90-48.60)	36.80 (23.40-47.30)	40.20 (21.30-50.30)	35.60 (14.90-49.50)
β2-microglobulin (mg/L)						
Median (range)		5.23 (1.08-160.20)	5.03 (1.08-160.20)	5.57 (1.73-80.00)	5.65 (1.80-32.42)	2.98 (1.53-22.19)
M protein (g/L)						
Median (range)		17.55 (0.00-167.80)	19.70 (0.00-167.80)	8.27 (0.00-76.50)	12.17 (0.00-119)	2.49 (0.00-19.68)
Cytogenetic risk disease						
High		57	39	4	14	——
Standard		107	84	5	18	——
Unknown		72	52	9	11	——

PCDs, plasma cell dyscrasias; MFI, mean fluorescence intensity; β2-M, β2-microglobulin; LDH, lactic dehydrogenase; M Protein, monoclonal protein; FISH, fluorescence *in situ* hybridization; ISS, Internal Staging System; R-ISS, Revised ISS; DS, Durie-Salmon.
