# Supplementary material for: CS1 Expression Pattern in NK Cells and Correlated Factors in Plasma Cell dyscrasias: Implications for Elotuzumab Therapy and CAR-T Efficacy
Source: J Cancer. 2024 Apr 8;15(10):3065–75. doi: 10.7150/jca.93637 (PMC11064268; doi:10.7150/jca.93637)
Supplement: Supplementary file 1 — Supplementary figures and table. [file jcav15p3065s1.pdf]

Supplementary Material

Supplemental Figures

Figure S1

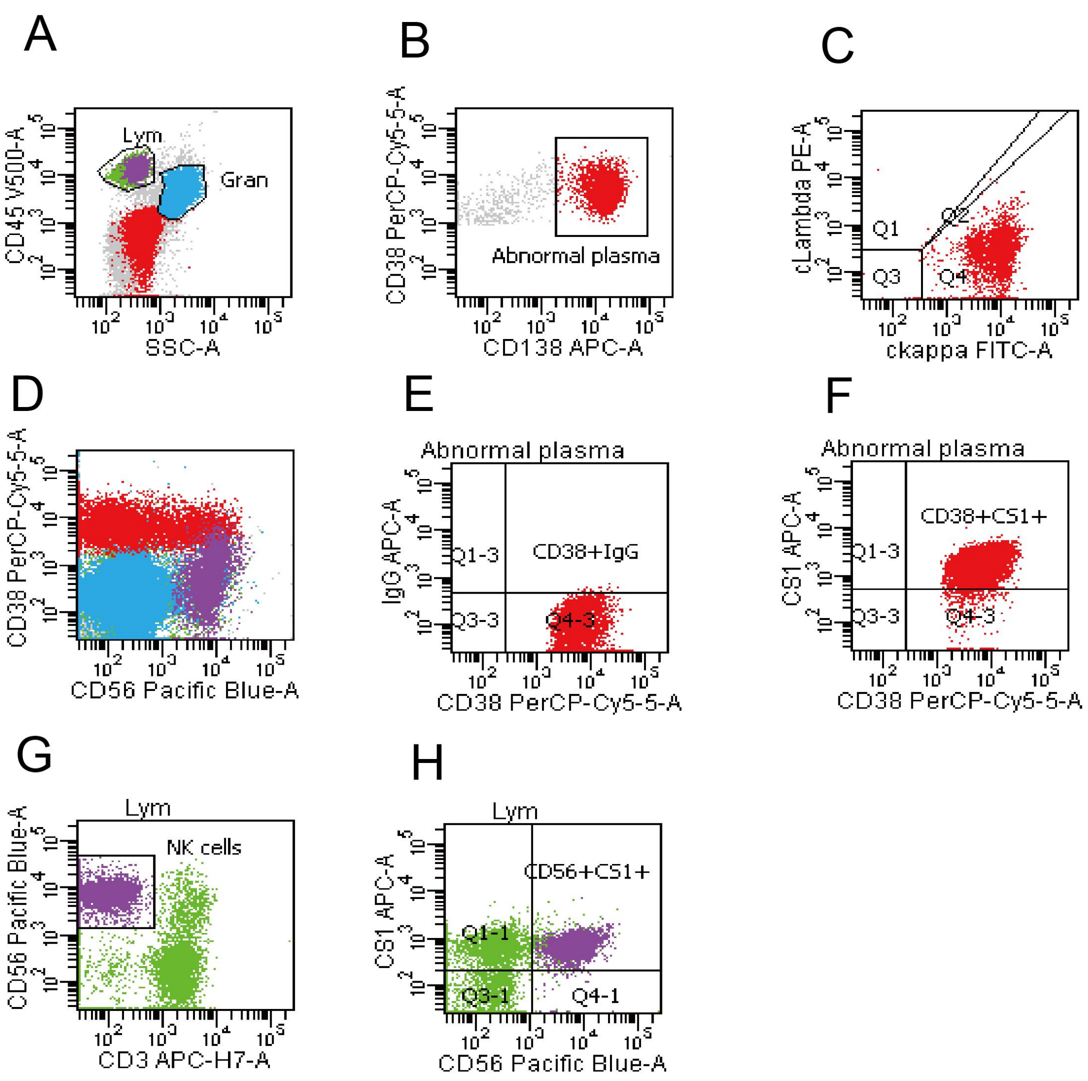

Figure S1. The detailed gated method of flow cytometry.

(A) The lymphocytes and granulocytes were gated by CD45 and SSC. (B-E) SSC/CD38/CD45 /CD138 /CD56, as well as ckappa and clambdas were used to gate the

abnormal plasma cells. (F) CS1 expression in these abnormal plasma cells (G-H) Within the lymphocyte gate, the NK cell population were gated by CD3⁻/CD56⁺, and then CS1 expression was analyzed in them.

Figure S2

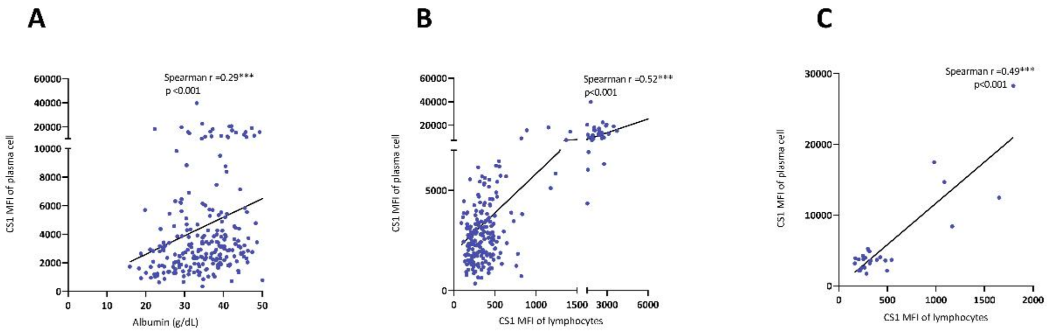

Figure S2. Spearman correlation analysis of CS1 expression of plasma cells and clinical parameters. (A)displayed Spearman relationships between serum albumin concentration and the CS1 MFI of plasma cells in MM. (B) and (C) displayed Spearman correlations between the CS1 MFI of lymphocytes and the CS1 MFI of plasma cells in MM and other PCDs respectively. ***:P<0.001.

Figure S3

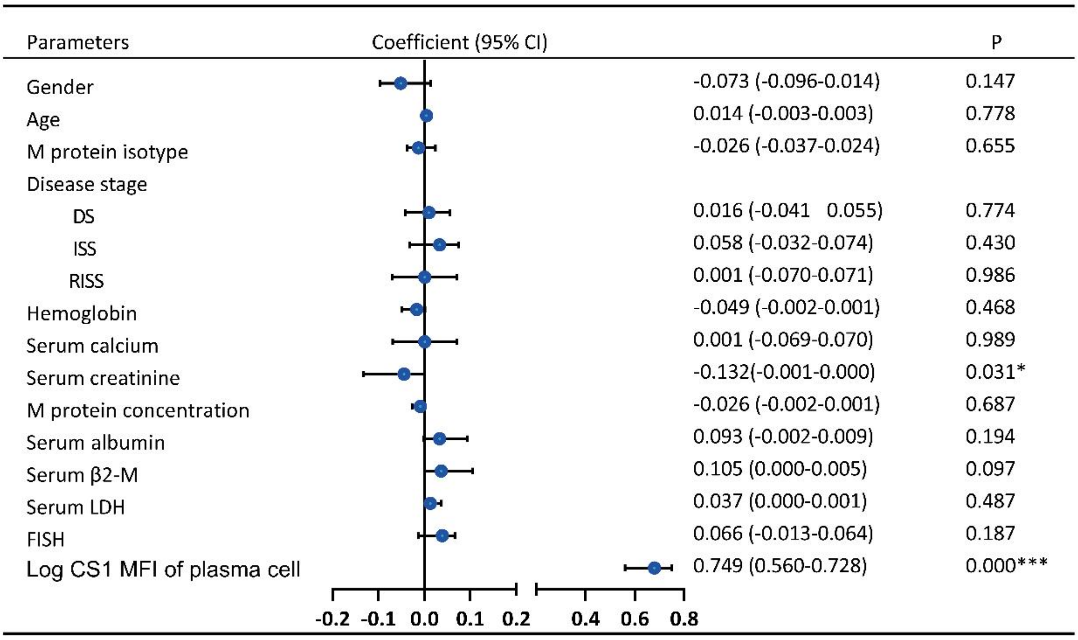

Figure S3. Multivariate Analysis for the CS1 expression of plasma cell in MM.

The forest plot shows the correlation between CS1 expression levels of plasma cells and clinical parameters through multivariate linear regression analysis. The expression level of CS1 in NK cells is independently associated with the expression level of CS1 in clonal plasma cells.

Figure S4

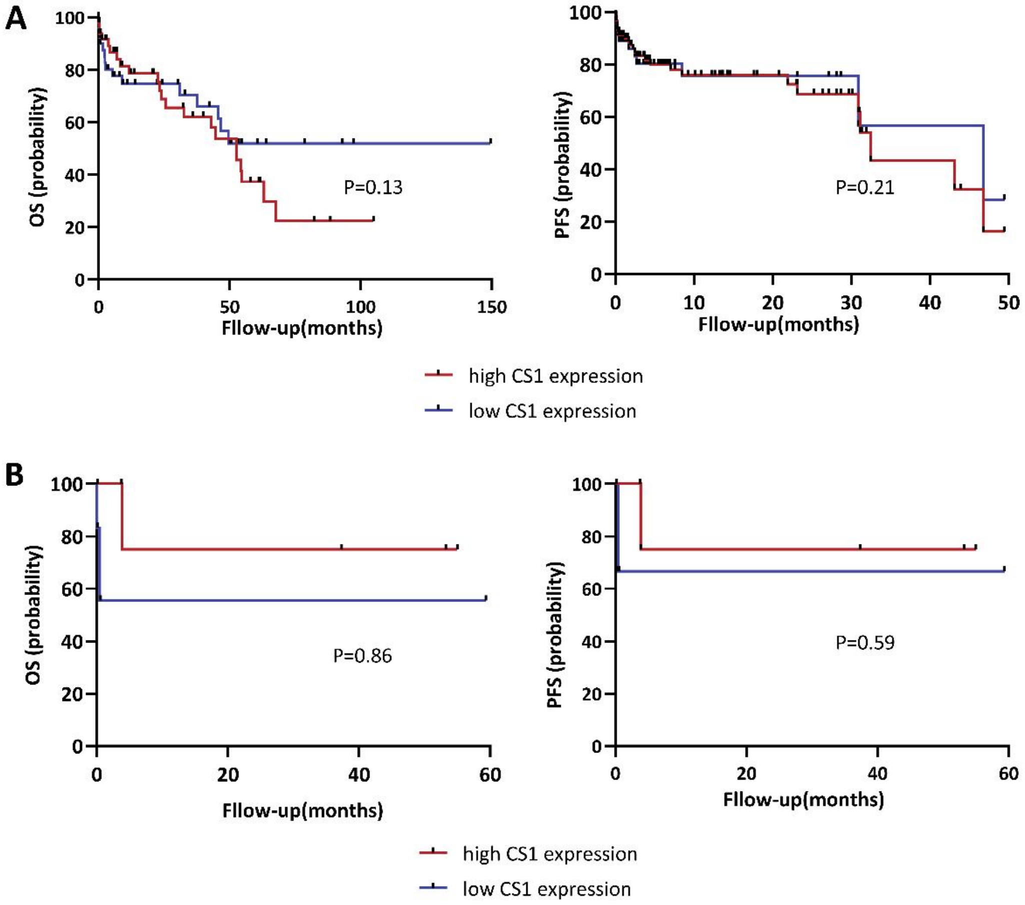

Figure S4. The correlation of CS1 expression level of plasma cells with PFS, OS.

(A-B) Comparative analysis the OS and PFS between patients with high and low CS1 expression of plasma cells in MM and other PCDs respectively. According to the expression of CS1 on monoclonal plasma cells, patients were divided into a high expression group (CS1 MFI ≥ the 75th percentile) and a low expression group (CS1 MFI ≤ the 25th percentile).

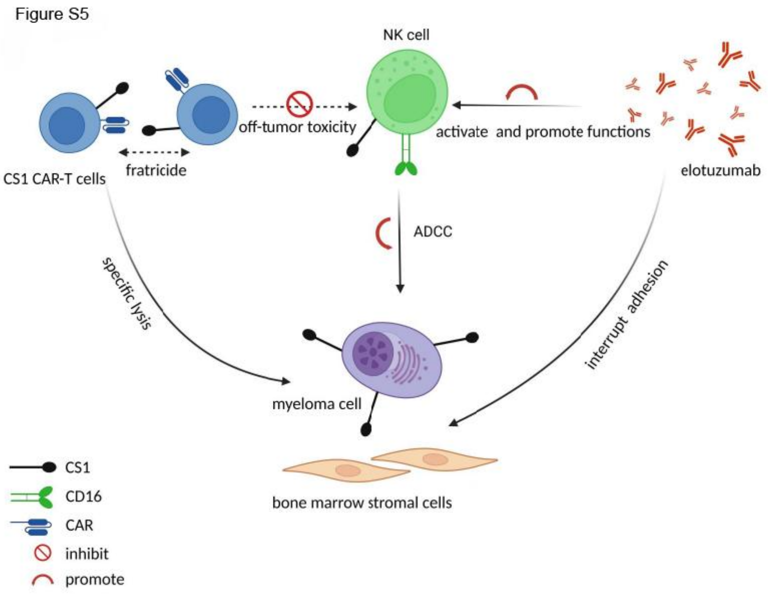

Figure S5. Expression of CS1 on NK cells has implications for immunotherapy targeting CS1. Elotuzumab relies on NK cells to induce myeloma cell lysis through ADCC and may also disrupt adhesion between myeloma cells and bone marrow stromal cells. Therefore, selecting a less pretreated patient and increasing the number and function of NK cells may be the convincible ways to promote the efficacy of Elotuzumab. Killing NK cells by CS1 CAR-T not only leads to excessive toxicity but also weakens the immunological effect of NK cells against MM. Therefore, avoiding killing NK cells by CS1 CAR-T helps improve safety and efficacy. This figure was created with BioRender.com.

Supplementary Tables

	OS		PFS	
	HR (95%CI)	*P*	HR (95%CI)	*P*
**Sex**	0.769(0.502-1.178)	0.227	0.954(0.668-1.362)	0.794
**Age**	1.032(1.009-1.055)	0.006^{**}	1.019(1.001-1.038)	0.041^{*}
**DS Stage 3**	1.074(0.605-1.907)	0.808	0.748(0.446-1.254)	0.271
**ISS Stage 3**	1.405(0.799-2.469)	0.237	1.208(0.748-1.95)	0.440
**RISS Stage 3**	0.607(0.305-1.208)	0.155	0.889(0.5-1.58)	0.688
**Hemoglobin**	0.995(0.984-1.005)	0.314	0.994(0.985-1.003)	0.180
**Calcium**	1.901(1.085-3.331)	0.025^{*}	1.368(0.875-2.139)	0.169
**Creatinine**	1(0.999-1.002)	0.867	1(0.999-1.001)	0.856
**M protein**	0.997(0.987-1.007)	0.539	1.001(0.992-1.01)	0.822
**Albumin**	0.988(0.946-1.031)	0.571	0.999(0.963-1.036)	0.966
**β2-M**	1.018(0.999-1.038)	0.070	1.026(1.008-1.044)	0.004^{**}
**LDH**	1.001(1-1.001)	0.208	1(0.999-1.001)	0.693
**High Risk genetic Abnormalities**	1.015(0.633-1.627)	0.951	0.976(0.650-1.464)	0.905
**Bone marrow plasma cells**	0.998(0.985-1.012)	0.796	1.006(0.994-1.018)	0.347
**CS1 MFI of natural killer cells**	1(1-1.001)	0.253	1(1-1.001)	0.102
**CS1 MFI of plasma cells**	1(0.999-1)	0.221	0.999(0.999-1)	0.172

Table S1. Cox multivariate analysis of PFS and OS in multiple myeloma patients

Abbreviations: ISS, Internal Staging System; R-ISS, Revised ISS; DS, Durie-Salmon; M Protein, Monoclonal protein; β2-M, β2-microglobulin; LDH, lactic dehydrogenase; MFI, mean fluorescence intensity; *: *p* values less than 0.05 (two-tailed) were considered statistically significant; **: *P<0.01*
